# Patches of Dysflective Cones in Eyes With No Known Disease

**DOI:** 10.1167/iovs.63.1.29

**Published:** 2022-01-24

**Authors:** Ethan Bensinger, Yiyi Wang, Austin Roorda

**Affiliations:** 1 Herbert Wertheim School of Optometry and Vision Science, University of California, Berkeley, California, United States

**Keywords:** adaptive optics, microperimetry, structure/function

## Abstract

**Purpose:**

To characterize the structure and function of patches of dysflective cones in the foveal region of subjects with normal vision and no known pathology. Dysflective cones are cones that have little or no reflective properties in optical coherence tomography (OCT) or adaptive optics scanning laser ophthalmoscope (AOSLO) images yet exhibit measurable function.

**Methods:**

AOSLO images were surveyed for the presence of hyporeflective cone patches, and subjects were brought back for imaging to determine the changes in the hyporeflective region. Adaptive optics microperimetry (AOMP) was used to assess the function of hyporeflective patches in four subjects to determine that they did, in fact, contain dysflective cones. AOMP utilized a stimulus size of less than 1 arcmin to measure thresholds inside and outside the hyporeflective region.

**Results:**

Nineteen out of 47 individuals retrospectively reviewed had one or more regions with hyporeflective cone patches in one or both eyes. Ten subjects with hyporeflective cone patches were brought back for imaging. Seven of the 10 had resolved at follow up, and in three subjects new hyporeflective patches appeared in a different location. All AOMP-measured subjects had measurable function in the dysflective cone region. Three out of four subjects showed no difference in light sensitivity in the dysflective region compared to adjacent areas, and one subject showed a 3× reduction in sensitivity in the area.

**Conclusions:**

Patches of dysflective cone have been identified in subjects with normal vision and no known pathology, and we have observed instances where dysflective cones in these subjects regain normal reflective properties.

Cone photoreceptors initiate human photopic vision, providing the critical first response to light. Their tiled and close-packed arrangement within the retina limit, in part, the spatial resolution of the eye. Due to their high metabolic demand, cones are very susceptible to disease. Visualizing cone structure in living eyes offers a way to detect and monitor retinal disease. The adaptive optics scanning laser ophthalmoscope (AOSLO) system uses adaptive optics to correct for optical aberrations in the eye and achieve near diffraction-limited imaging of the individual photoreceptors at their densest packing in the fovea.^1^^–^^3^ This high-resolution retinal imaging capability can be used to assess the health of cone photoreceptors by measuring the intrinsic ability of the cones to reflect light with confocal AOSLO systems.

Cone reflectivity measured in the confocal AOSLO can be a challenging metric for retinal health due to significant spatial and temporal variations.^4^ Intra-cone reflectivity changes over time could be due to a multitude of factors, including light interference effects,^5^^,^^6^ outer segment length changes due to disc shedding,^7^ pigment density changes,^8^ and optical waveguiding.^9^ Inter-cone differences in reflectivity, however, are unrelated to cone type except under carefully controlled conditions,^10^ and local reflectance angle differences are unlikely causes for large reflectivity differences.^11^ Additionally, cone reflectivity has not been found to be related to the reflectivity of surrounding cones, and individual hyporeflective cones have shown normal visual function.^12^

Despite the sources of cone reflectivity variability described above, it is generally the case that regions in AOSLO and optical coherence tomography (OCT) images that exhibit a lack of normal reflections from the photoreceptors correspond to regions that lack functional cones. Multiple publications have confirmed this for many different diseases, including retinitis pigmentosa,^13^^,^^14^ choroideremia,^15^^,^^16^ cone–rod dystrophy,^17^^,^^18^ age-related macular degeneration,^19^ achromatopsia,^20^ oligocone trichromacy,^21^ fundus albipunctatis,^22^ and Stargardt disease.^23^^,^^24^

However, the absence of visible cones in a retinal image (i.e., hyporeflective cones), even over large areas, does not necessarily mean that cones are not present or that they are not functional. A study of a patient with acute bilateral fovealitis found measurable sensitivity and visual acuity within a retinal region that had no apparent cones in the confocal AOSLO image or in a spectral-domain OCT image, a phenomenon that was termed by Tu et al.^25^ *dysflective cones.*^25^ Dysflective cones are a specific subclass of hyporeflective cones. They are hyporeflective but also retain measurable function. In the case of Tu et al.,^25^ the dysflective cones appeared dark in confocal AOSLO images and, in OCT images, the region of the B-scan normally occupied by reflective layers corresponding to the inner and outer segment (IS/OS) junction and the cone outer-segment tips were transparent, although there was an intact external limiting membrane (ELM). Similarly, cones in a dysflective state were observed by Wang et al.^26^ in two patients with macular telangiectasia.

Importantly, Wang et al.^26^ found that regions where cones appeared to be absent in one visit recovered normal reflective properties in a subsequent visit. The recovery of reflective properties in cones of patients with macular telangiectasia has also been confirmed in a more recent and extensive study that employed both confocal and split-detector AOSLO imaging.^27^ Similar recovery has also been observed in OCT images in patients following macular hole repair.^28^^,^^29^ None of the these three reports, however, was able to directly test the function of the purported dysflective cones. Litts et al.^27^ also discovered that, although cones may not appear in confocal AOSLO or OCT images, the inner segments of the cones *do* appear when using split-detector AOSLO imaging. Unlike AOSLO and OCT, both of which employ confocal imaging, split-detector AOSLO is a form of phase-contrast imaging and does not rely on direct backscattering for detection and resolution of cell structure.^30^^,^^31^

When imaging volunteer subjects in the AOSLO that self-report to have normal vision and no known pathology, in addition to the normal cone-to-cone variability, we frequently observe small hyporeflective patches in the cone mosaic. These hyporeflective patches have a similar appearance to clusters of dysflective cones seen in patients and are also transient, both appearing and recovering normal reflectivity similar to dysflective cones in patients with macular telangiectasia type 2.^26^^,^^32^ Thus, we were motivated by these observations to investigate the frequency of occurrence and timelines of hyporeflective patches in these eyes and to evaluate function within these patches. We hypothesize that these hyporeflective patches contain dysflective cones (i.e., they retain function). The AOSLO system used in this study has been equipped with real-time eye tracking; it has the ability to deliver light to targeted retinal locations^33^ and offers an unprecedented ability to test function in regions as small as a single cone.^34^

## Methods

### Human Subjects

The experiments were approved by the institutional review boards at the University of California, Berkeley (UCB), and the University of California, San Francisco (UCSF), and research procedures followed the tenets of the Declaration of Helsinki. We excluded all subjects who were under 18 years of age and women who were pregnant and/or nursing. Informed written consent was obtained from all eligible subjects after explanation of the nature and possible consequences of the study, including the possible use of their images and data for retrospective review and ancillary studies. Part of the informed consent process was to ask subjects to self-report any difficulties with their vision or any known pathology or ocular conditions that might preclude imaging. Nothing remarkable was reported in this cohort. Finally, subjects with high refractive errors that might preclude successful imaging were excluded. This cohort did not include any subjects with myopic refractive errors greater than –8 diopters.

### AOSLO Imaging

AOSLO images of the cone mosaic were acquired using multiwavelength AOSLO platforms at both UCB and UCSF, the details of which have been described previously.^34^^–^^36^ Both systems operate with identical control software, have a similar optical design, and were designed and built by members of Roorda's laboratory. In these systems, the light source was a supercontinuum laser (SuperK EXTREME; NKT Photonics, Birkerod, Denmark) that was divided into multiple channels using a series of custom-built fiber couplers to provide an 840-nm (12-nm bandwidth) channel for infrared imaging, a 680-nm (22-nm bandwidth) channel for visible red imaging, and a 543-nm (22-nm bandwidth) channel for visible stimulation. The 543-nm stimulus delivery was chosen for functional testing because it was equally sensed by L and M cones. The system measured wave aberrations with a custom-built Shack–Hartmann wavefront sensor and employed a deformable mirror with 97 actuators (DM97; ALPAO, Montbonnot-Saint-Martin, France) to compensate the aberrations.

### Retrospective Survey of Hyporeflective Cones in Eyes with No Known Pathology

Images previously collected from the AOSLO systems at UCSF and UCB were surveyed. The AOSLO raster was set to 0.9° × 0.9° (∼270 × 270 µm) with an average sampling resolution of 9.48 pixels/arcmin. Ten-second videos were recorded with 680-nm visible red light for the majority of imaging, but some subjects were recorded at 840 nm. Videos were recorded at nine locations at and around the fovea representing a minimum 1.8° × 1.8° area. The survey included images from 74 eyes of 47 subjects (22 male, 25 female). The average age for these subjects was 28.4 years, with a maximum age of 52 years and minimum age of 20 years. The primary purpose for imaging 62 of the eyes (84% of the study) was for a study on foveal cone spacing as a function of axial length.^3^ The remaining 12 eyes were imaged for various other studies. In some cases, an image from a single video of an ∼0.9° field centered on the fovea was used.

### Tracking Hyporeflective Cone Progression

Suspected dysflective cones were identified as contiguous hyporeflective patches in the cone mosaic with no discernible cones. Patches encompassing areas that would have been occupied by as few as three hyporeflective cones were included in this report. In addition to the subjects used in the retrospective survey, seven new subjects were recruited for imaging following the same imaging protocol as Wang et al.^3^

Whenever possible, the subjects from both the retrospective study and newly recruited subjects with patches of suspected dysflective cones were brought back for follow-up imaging. Follow-up imaging was always done on the same AOSLO system at either UCB or UCSF. Two subjects from the retrospective study already had follow-up imaging done, and eight additional subjects were brought back specifically for the purposes of this study. To ensure that images were recorded from the same retinal location on repeated visits, subjects were asked to fixate on landmarks within the scanning raster (e.g., corners of the square) or a fixation cross on a display that was presented to the imaged eye via a beamsplitter in the AOSLO optical path.

For all new subjects, we verified that the hyporeflective patches were not due to shadows from inner retinal layers by performing a through-focus confocal imaging sequence to search for any shadow-causing disruptions in the anterior to the photoreceptors. This test ruled out one potential subject, as their hyporeflective patch was caused by a shadow from a small inner-retinal cyst.

### Analyzing Hyporeflective Patches

The raw AOSLO videos collected in this survey were converted to high-resolution, high-signal-to-noise images first by correcting for eye movements offline using custom MATLAB (MathWorks, Natick, MA, USA) software and then adding all registered frames.^37^ Briefly, the eye movement correction process involves breaking each frame of the 680-nm or 840-nm acquired video into strips and cross-correlating them to a reference image. The reference image was created by cross-correlating then summing multiple frames of the video together to obtain a large reference image with less eye-motion distortion than a single frame. For eyes with the nine imaging locations representing a 1.8° × 1.8° field, each image was cropped and assembled to form a montage (Photoshop; Adobe Inc., San Jose, CA, USA), and the dysflective regions were identified in these montages.

Whenever suspected patches of dysflective cones were identified, images were histogram normalized to a reference image, and the border of the hyporeflective region was calculated using the fast-marching method.^38^^,^^39^ The border was measured by selecting a pixel in the dysflective region to use as a reference intensity and then computing the weight for each pixel in the image based on the difference between the grayscale intensity of each pixel and the reference. The image was then segmented using the fast-marching method with a consistent threshold and the reference pixel as the seed location.^38^^,^^39^ Following that, any holes within the segmentation area were filled, and the perimeter of the segmented area was calculated.

### Functional Testing

Adaptive optics microperimetry (AOMP) was used to assess the function of areas identified as possible dysflective cones in four healthy subjects. The AOSLO can test the function of the retina with cellular accuracy by measuring and correcting retinal image motion in real time and delivering an AO-corrected, stabilized stimulus directly to a targeted retinal location.^33^ Previous reports from our laboratory confirm that the AOSLO system can target individual cones and assess their sensitivity with AOMP.^34^^,^^40^

In this study, AOMP thresholds were computed as the average of two 35-trial adaptive staircase (QUEST) sessions.^41^ Adjacent normal-appearing mosaics of cones were used as a control. Due to the small size of the hyporeflective patches, AOMP utilized a stimulus size of less than 1 arcmin. Transverse chromatic aberration (TCA), due to the difference in the imaging (840 nm) and testing (543 nm) wavelengths, was measured objectively before and after AOMP testing.^42^ During the trials, a pupil camera was used to ensure that the eye remained well aligned in the system so that TCA would not change during the trials by more than 0.5 arcmin.^43^ Each trial was comprised of a stimulus that was delivered over six frames (∼150 ms). A 1-s AOSLO video, which contained a digital mark indicating the location of the stimulus on each frame, was recorded for each trial.

The exact location of the tested region for each threshold measurement is affected by several factors:

1. Due to real-time tracking errors, the stimuli are not delivered to the same exact location of the retina on each frame of each trial. All trials where the delivered stimulus in any one of the six frames was off by more than 20 pixels (∼2 arcmin) were removed from the QUEST staircase. The remainder of the stimulus locations formed a tight scatterplot of tested locations about the targeted area.
2. After the AO correction, the focused point spread function (PSF) is still blurred by diffraction. We approximated the final PSF after correction to be a diffraction-limited Airy pattern.
3. The stimulus itself has a finite size that is set by the operator. In this experiment, that stimulus was a square patch less than 1 arcmin in size.

Considering the three factors described above, the accuracies of the targeted locations in a complete AOMP session were depicted by convolving the stimulus with the diffraction-limited Airy pattern and summing over the complete range of locations where the stimulus was delivered over all trials. A contour map was then generated to indicate the average distribution of light delivered to the retina on a normalized percentage scale.

Thresholds were computed on a 1000-step normalized scale from 0 to 1 (30-dB range). Lower thresholds indicate higher sensitivity and a value of 1 indicates that the maximum amount of power that we could deliver in any session was not seen. For these particular measurements, a value of 1 corresponded to a maximum luminance of 38 cd/m^2^. The background was dominated by the 840-nm imaging channel, and, when combined with the leak in the 543-nm stimulus channel, our background was on average about 4 cd/m^2^. Thresholds are reported here in normalized units (*I_t_*), but they can be converted to sensitivity on a decibel scale (*S*_dB_) using the following formula:

$$S_{\mathrm{dB}}=10\;\times \log_{10}\left(1/I_{t}\right)$$

At this light level, rods should be saturated, and within the 1.8^○^ field there should be few if any rods in the area.^44^

## Results

### Survey of AOSLO Images

**Figure 1.**
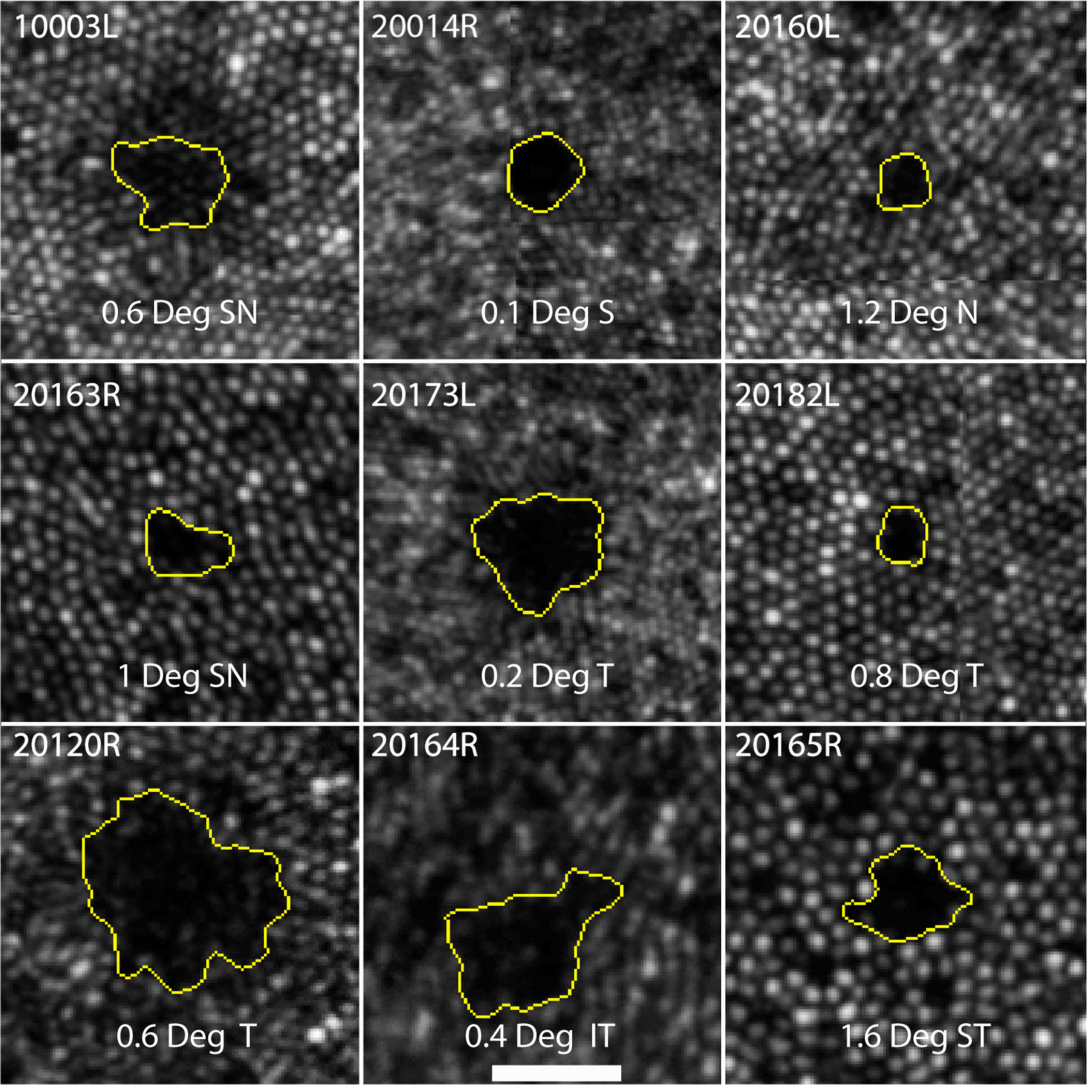
Hyporeflective cone clusters from nine subjects. *Yellow borders* were created using the fast-marching method. Lines that appear within some of the panels are due to small alignment mismatches at the borders between the individual image frames that comprise the montage. The *scale bar* in the lower central panel is 0.1° and applies to all frames.

In our retrospective study, confocal AOSLO images revealed patches of suspected dysflective cones in distinct areas in the foveal region in many of our subjects, none of whom had any known ocular pathology. We found that 19 out of the 47 individuals had one or more hyporeflective patches in one or both eyes. Example images of these hyporeflective patches are provided in Figure 1, shown in linear scale. A logarithmic scaling of the images revealed that some reflectivity is often present within these patches, but a clear and contiguous mosaic consistent with cones was never visible.

For these retrospective cases we did not focus anterior to the hyporeflective patches to rule out shadows from the inner retina, so we are not able to rule that out completely as a cause. We feel that shadows from blood vessels, however, are highly unlikely because of their round (not linear) appearance and they were within the foveal avascular zone.

The areas are generally close to circular, with an average circularity index (ratio of area to perimeter) of 0.73 with a standard deviation of 0.029.^45^ The average area of the hyporeflective patches was 6.3 arcmin^2^ (range, 2.2–57.3 arcmin^2^). There was no noticeable tendency for the hyporeflective patches to be appear in any specific direction relative to the fovea. The average eccentricity and range of the hyporeflective patches relative to the fovea was 0.66° (range, 0.24°–1.46°), but this range of eccentricities reflects a sampling bias because we only looked at images of the foveal region.

**Figure 2.**
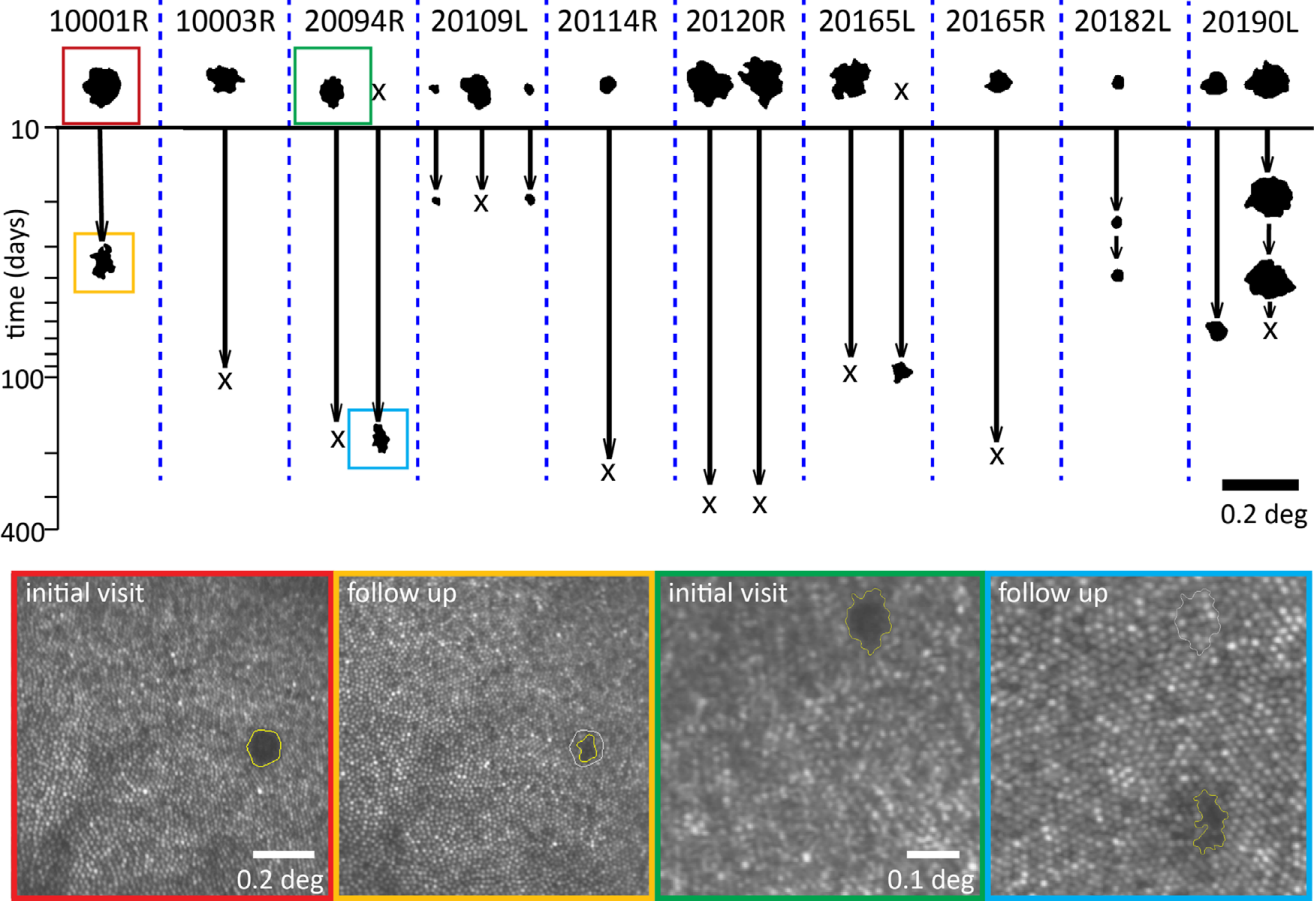
Timeline of hyporeflective patches in 10 eyes of nine subjects. The *y*-axis is the time in days since their original sighting. An X at follow-up indicates that the area resolved over time, and an X at the original imaging session indicates that a new hyporeflective patch was seen at follow-up. The images corresponding to the patches inside the *colored boxes* are shown in the *lower panels*. Subject 10001R showed partial recovery of reflectivity over 5 weeks. Subject 20094R had complete recovery of one hyporeflective patch, with a new hyporeflective patch appearing in a new location 5.25 months later.

**Figure 3.**
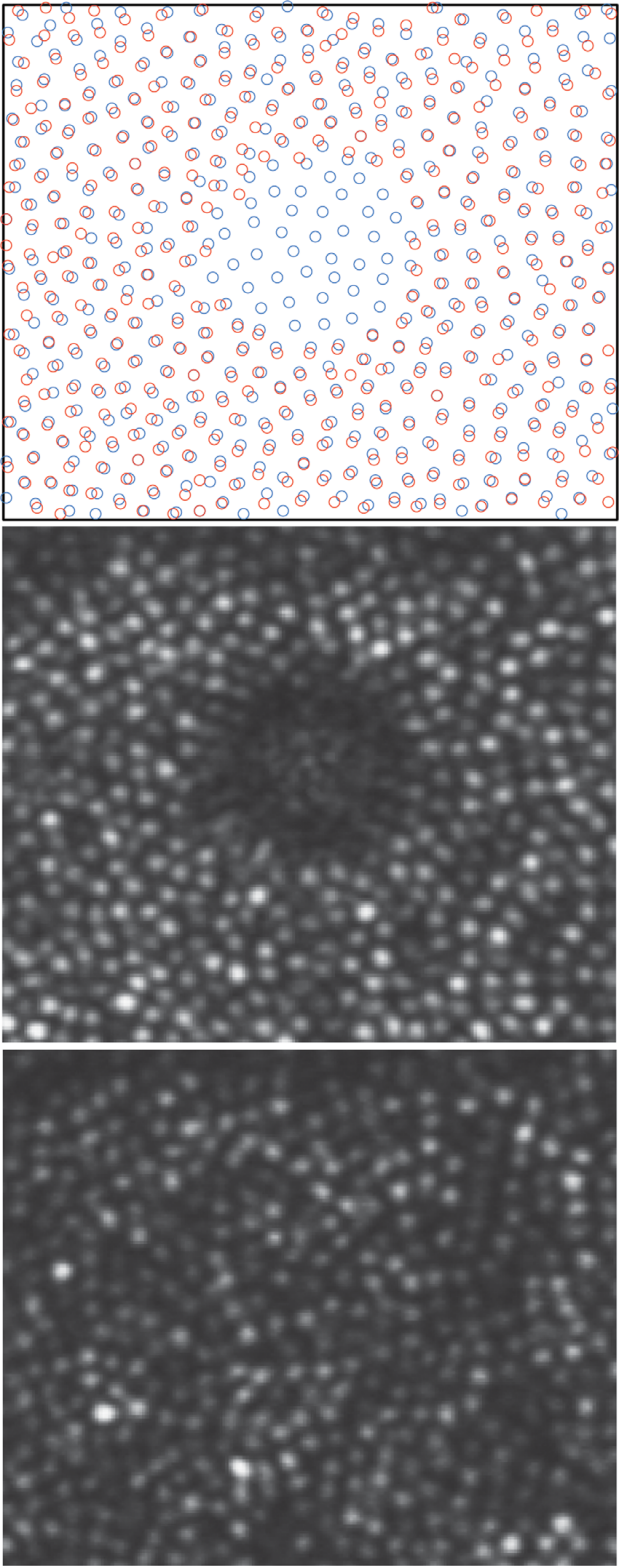
Cones do not migrate into the hyporeflective regions. Cone locations from subject 20190 from the initial visit (*middle* *panel*) are indicated in the *upper* *panel* by the *orange circles*. The initial selections surround the central hyporeflective patch where no cones were visible. The *blue circles* indicate the cone locations at follow-up (*lower* *panel*). There is a nearly perfect correspondence between the original and final cone locations. The only change is the new selections of the cones that regained their reflective properties at follow-up.

Whenever possible, images of the same region taken on different days were compared. Time spans for follow-up imaging ranged from 3 weeks to 1 year. The areas for the 10 subjects with repeated imaging sessions are shown in Figure 2. Of the 14 hyporeflective patches in 10 of the subjects that were present at the first imaging session, nine fully resolved at follow up, denoted by an Χ at the lower terminus of the timelines. We were not able to measure the exact time point when the hyporeflective patches appeared or resolved. New clusters spontaneously appeared in other retinal locations in two eyes from two subjects and are denoted by an X at the baseline. Areas that recover reflectivity were verified not to result from cones migrating to that space by performing a cone-to-cone match between the two visits. To illustrate this, Figure 3 shows examples from one subject where the initial cone locations surrounding the hyporeflective patch are shown in orange and the cone locations in the follow-up visit are shown in blue.

### Functional Testing Reveals That Hyporeflective Patches Contain Dysflective Cones

**Figure 4.**
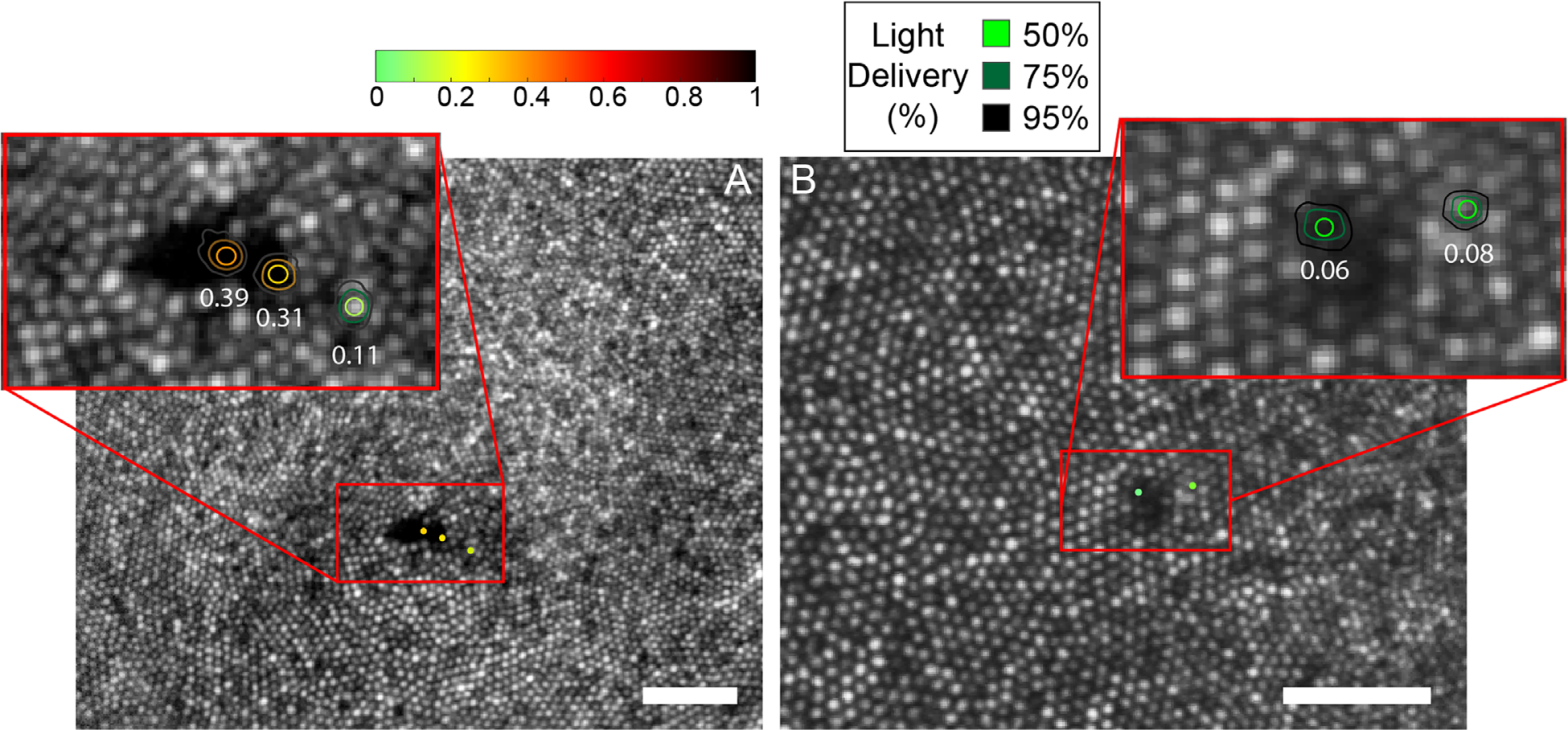
Microperimetry results for subjects 40104 (*left*) and 20182 (*right*). The tested locations are indicated by *dots* in the larger field image and by *contours* in each magnified inset where the *color* of the *dot* and the central contour depicts the threshold as indicated by the *color* *bar*. The *scale bars* in each image are 0.2°.

**Figure 5.**
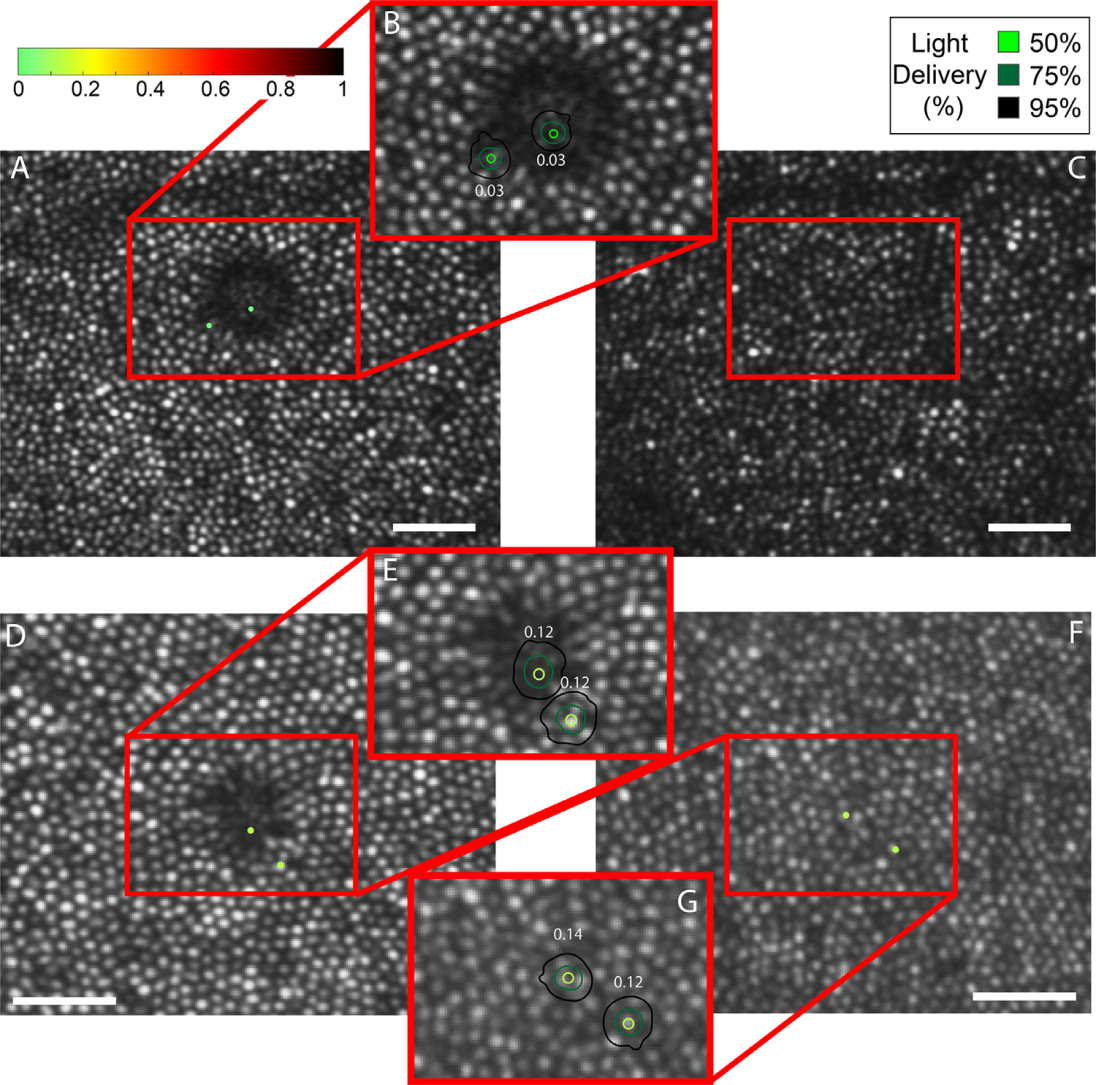
Microperimetry results for subject 20190 (*top*) and 20114 (*bottom*). The tested locations are indicated by *dots* in the larger field image and by *contours* in each magnified inset where the *color* of the *dot* and the *central contour* depicts the threshold as indicated by the *color* *bar*. Panels (**C**) and (**F**) to the right show the follow-up AOSLO images (3 weeks for 20190 and 2 weeks for 20114) after the first microperimetry session where the dysflective cones have recovered their reflectivity. Here, the *red box* indicates the same location as the *red box* in (**A**) and (**D**). (**F**) also shows the microperimetry results in the location with recovered reflectivity. The *scale bars* in each image are 0.1°.

Light-sensitivity thresholds within hyporeflective patches were measured in four subjects, and the results are shown in Figures 4 and 5. The targeting accuracy for all subjects is indicated by contour plots at each test location, computed as described earlier. TCA values were measured before and after each session and were confirmed to be within 0.55 arcmin in both the *x* and *y* direction of each other for all subjects.

All subjects had measurable sensitivity in their hyporeflective patches, thereby confirming our suspicion that they indeed contained dysflective cones. Three of the four subjects showed no differences in sensitivity within their hyporeflective patches compared with adjacent areas with normal appearing cones. One subject (40104) had 3× reduced sensitivity within their hyporeflective patch, as shown in Figure 4.

Cones within two of the four subjects’ hyporeflective patches regained normal reflective properties after 2 to 3 weeks, as shown in Figure 5. We had the opportunity to perform AOMP in one of the subjects (subject 20114) (Fig. 5, lower panels). The thresholds were similar before and after recovery of reflectivity.

## Discussion

Patches of hyporeflective cones were common in the subjects with normal vision and no known pathology (19 of 47). As our study was limited to a retrospective search in the foveal area only, this number should be considered a lower bound on their prevalence. In four out of the four subjects studied with AOMP, these hyporeflective patches of cones had similar or slightly reduced function compared with the surrounding cones, confirming that they were indeed dysflective cones. It is reasonable to consider that most, if not all, of the hyporeflective patches in the other 15 subjects would have also been confirmed to be dysflective. We also show that dysflective cone patches in these eyes are transient, both resolving and appearing in new locations over time.

### The Causes for Dysflective Cones Remain Unknown

The reason why cones go into and recover from a dysflective state is yet unknown. Reflected light from cones in retinal images originate from two primary reflection sites within the cone: the IS/OS junction and the cone outer segment tips. These reflection sites are readily visible in OCT images, which visualize sources of reflection by their time of flight.^46^^,^^47^ In an AOSLO, the two reflections combine and are visualized at the point where they emerge from the inner segments near the ELM.^5^^,^^48^ Because the two sources of light combine in an AOSLO, there is a possibility that the cone reflectivity is reduced because of destructive interference between the two sources within the waveguiding cones.^5^^,^^48^^,^^49^ There are two reasons why interference is an unlikely cause for cones to be in a dysflective state. First, the hyporeflective patches persist for some time, whereas interference effects tend to change rapidly.^50^^,^^51^ Second, the relatively wide bandwidths of the imaging sources (11 nm for 840 and 22 nm for 680) give rise to coherence lengths (∼21 µm and ∼ 7 µm, respectively)^52^ that are shorter than the twice the length of the outer segments, thereby minimizing the amount of interference that can occur.^5^^,^^49^

Ruling out interference, then it must be the case that both sources of reflection within a dysflective cone are diminished. This is indeed observed in OCT images.^25^^,^^26^ Changes in the reflectivity of both sources could arise from changes in the pointing direction of the cone.^53^ Some evidence of local cone directionality changes has been reported around drusen^54^ and even eye diseases with no obvious topographical changes.^55^ However, we feel that abrupt local changes in pointing direction that are large enough to effectively eliminate all reflections for such small patches of close-packed cones in eyes with no known disease are unlikely. Physical factors force cones to line up with their neighbors.^11^^,^^56^ Furthermore, directional OCT assessment of a dysflective patch of cones in a previous paper authored by members of this author group revealed no evidence of misdirected cones.^25^

We are left to conclude, then, that a structural change in the outer segment giving rise to a reflectance change is the most likely cause. The persistence of light sensitivity, however, implies that phototransduction is still occurring. Some evidence of a decoupling between function and reflectance of cone photoreceptors can be found in a study on non-human primates^57^ where, 90 days following an intentional retinal detachment with a bleb of balanced salt solution, the function (measured by multifocal electroretinography) recovered completely yet the reduced reflectivity of the IS/OS junction persisted. Electron microscopy of the cones revealed that those weak IS/OS reflections were associated with irregular and large intradiscal spaces in the affected outer segments, distinct from the regular, tightly packed discs in a typical outer segment.

Unfortunately, alternative imaging methods were not able to provide additional structural details of these dysflective cones. Commercially available spectral-domain OCT systems did not offer the sampling density or resolution to resolve these hyporeflective patches, as it was unclear which layer the reduced reflectivity was within in the outer retina. We also attempted to visualize the cone inner segments using split-detection AOSLO,^30^ which is a form of phase-contrast imaging that reveals cells by their refractive state rather than their reflective properties. However, notwithstanding recent developments,^58^ split-detector imaging has inherently poorer resolution than confocal imaging, and the cone inner segments near the fovea in our subjects were too small to resolve using that modality. Notably, other studies have used split-detector imaging to visualize inner segments in non-reflective cones away from the foveal center.^13^^,^^27^^,^^30^^,^^59^ Given that we measured function even in regions where we failed to see evidence of photoreceptor cells, and that in many instances we observed a full recovery of reflectivity, it is highly likely that these dysflective areas contained a complete mosaic of cone photoreceptor inner segments.

### Comparison With Previous Reports

The current results are consistent with a report from Bruce et al.,^12^ who found normal function in individual weakly reflective photoreceptors. In that study, individual photoreceptors were tracked in an AOSLO; some were found to have intermittent recovery of reflectivity but others remained persistently hyporeflective over as long as 672 days. The function of those individual weakly reflective photoreceptors was measured using a similar AOMP thresholding paradigm and the same 543-nm stimulating wavelength, and it was found that both persistently hyporeflective cones and intermittently hyporeflective cones had no detectable difference in threshold from normal cones. Despite the similar findings, the current study is distinct in that we chose to study hyporeflective patches rather than individual hyporeflective cones. Whereas the normal variations in reflectivity of a single cone could arise from a multitude of factors, these normal variations do not explain the larger patches of hyporeflective cones that we observed over multiple imaging dates.^4^^,^^5^^,^^12^^,^^49^

The dysflective cones that we observe in normal eyes have a phenotype similar to that of two clinical conditions previously reported: (1) a case-study report of dysflective cones in a patient with acute bilateral fovealitis,^25^ and (2) a study of subjects with macular telangiectasia who had dysflective cones that recovered reflectivity at the second time point.^26^

## Conclusions

Small patches of dysflective cones are relatively common in the retinas of subjects with normal vision. These dysflective cones are not associated with any known pathology. They exhibit normal or slightly reduced sensitivity, and in many cases they are transient, appearing and resolving over time.
